# Mechanistic insights into postoperative delirium using untargeted high-throughput proteomics in elderly patients - a case-control study

**DOI:** 10.1038/s41598-025-33931-6

**Published:** 2026-02-05

**Authors:** Mario Lamping, Maria Heinrich, Vadim Farztdinov, Clarissa von Haefen, Jayanth Sreekanth, Michael Mülleder, Markus Ralser, Georg Winterer, Claudia D. Spies

**Affiliations:** 1 Department of Anaesthesiology and Intensive Care Medicine (CCM, CVK), Charité - Universitätsmedizin Berlin, Augustenburger Platz 1, Berlin, 13353 Germany; 2 Berlin Institute of Health (BIH), Anna-Louisa-Karsch-Straße 2, Berlin, 10178 Germany; 3 High-Throughput Mass Spectrometry Core Facility, Charité - Universitätsmedizin Berlin, Charitéplatz 1, Berlin, 10117 Germany; 4 Department of Biochemistry, Charité - Universitätsmedizin Berlin, Charitéplatz 1, Berlin, 10117 Germany; 5 Pharmaimage Biomarker Solutions GmbH, 245 First Street, Cambridge, MA 02142 USA; 6 Charité - Universitätsmedizin Berlin, Charitéplatz 1, Berlin, 10117 Germany

**Keywords:** Proteomics, Mass spectrometry, Delirium, Classifier, Pathway analysis, Translational research, Proteomics, Predictive markers, Psychiatric disorders

## Abstract

Postoperative delirium (POD) in elderly patients is a serious complication after surgery with an unclear molecular pathogenesis. Perioperative untargeted high-throughput proteomic profiling may provide insights into underlying mechanistic molecular patterns and help identify patients at high risk, guiding prevention and therapy. This study is a monocentric substudy of the European BioCog project, a prospective multicentre observational study involving elderly patients aged ≥ 65 undergoing elective surgery with a Mini-Mental State Examination score of ≥ 24. POD was assessed twice daily for up to 7 days using the Nursing Delirium Screening Scale (Nu-DESC) and the Confusion Assessment Method for the Intensive Care Unit (CAM-ICU). We conducted high-throughput liquid chromatography mass spectrometry on sequential pre- and postoperative plasma samples. Data are available via ProteomeXchange with the identifier PXD071265. Data were analysed using a matched case-control design, employing both cross-sectional and longitudinal approaches, along with pathway enrichment analysis. A total of 226 highly abundant proteins were investigated in 168 patients (50% POD incidence). Multiple pathways, particularly those involved in the activation of the innate immune response and the complement system, were associated with POD. Butyrylcholinesterase showed the most robust regulation, with preoperative downregulation and postoperative upregulation in patients with POD, whereas it was further downregulated postoperatively in those without POD. Catalytic enzyme activity showed significant decrease in both groups post-surgery. A combined set of 8 preoperative proteins classified patients with POD with 86% sensitivity and 79% specificity. Untargeted high-throughput proteomics is a feasible approach to characterise pathways involved in POD pathogenesis. This case-control study identified a protein signature associated with POD, emphasising the need for larger cohorts to confirm these observations and improve the mechanistic understanding of POD.

**Supplementary Information:**

The online version contains supplementary material available at 10.1038/s41598-025-33931-6.

## Introduction

Postoperative delirium (POD) is a serious complication after surgery and anaesthesia^1^. Incidence rates vary considerably across studies, ranging from 5.1% up to 53.3% in a meta-analysis of 26 studies^1,2^, with highest incidences among elderly patients^3^. POD is related to increased morbidity and mortality, loss of autonomy, health care dependency, reduced quality of life, long-term neurocognitive disorders (NCD), depression and posttraumatic stress disorder^1,3^. Additionally, immense healthcare expenses result in significant socioeconomic effects^3^. Predisposing factors are numerous and include age, frailty, cognitive impairment and organ dysfunction, leaving the patient vulnerable to precipitating factors^4^. Among others, these include anaesthesia related factors (e.g. EEG burst suppression, drugs with anticholinergic side effects), surgical trauma, duration of anaesthesia and preoperative fasting^1,3,5^. Suspected pathomechanisms are multifactorial and remain poorly understood. Molecular mechanisms are hypothesised to involve neuroinflammation, neurotransmitter and neuroendocrine dysregulation, network disconnectivity, mitochondrial and metabolic dysfunction, oxidative stress, autophagy, cellular aging and circadian rhythm disruption^6^. We postulate that POD arises from multiple etiologies linked to acute encephalopathies triggered in predisposed patients, resulting in an “acute on chronic” disease model^3,4^. We propose these to be categorised in 4 domains:

(i) Toxicity axis: anaesthesia-related toxicity, anticholinergic perioperative medication.
(ii) Inflammatory axis: immune and complement activation, blood-brain barrier (BBB) disruption and neuroinflammation, endothelial dysfunction.
(iii) Metabolic dysregulation axis: energy metabolism, mitochondrial dysfunction, oxidative stress, autophagy imbalance.
(iv) Hypoxia axis: anaemia, hypoperfusion/hypotension, haemostatic dysregulation and coagulopathy/thrombosis.

Proteomics offers direct insights into (patho-)physiology underlying complex diseases such as POD, facilitating identification of therapeutic targets and associated markers and signatures^7,8^. Multi-protein signatures have been shown to increase sensitivity, specificity and the strength of prognostic and molecular stratification^7,9^. Recent advances in the field of high-throughput liquid chromatography and mass spectrometry (HT-LCMS) allow for a cost-effective analysis of the proteomic profile, especially for a large number of samples^9^. Several candidate proteins (e.g. C-reactive protein (CRP), tumour necrosis factor-alpha (TNF-ɑ), cholinesterase (ChE) activity) have been investigated in the context of POD but showed limited specificity and have not demonstrated clinical applicability to date^3,10,11^. MS has uncovered several potential molecular alterations associated with key pathological processes in POD, including neuroinflammation, oxidative stress, and synaptic dysfunction^3,6^. Among these, proteins such as cytokines (e.g., IL-6, IL-8, TNF-ɑ), neurotrophic factors (e.g., IGF-1), and acute-phase proteins (e.g., CRP, S100B) have been identified as promising candidates for diagnostic and prognostic applications^8,11^. However, studies are limited by unstandardised designs and methods, targeted analyses and small sample sizes^8,11,12^. This study aims to (i) compare perioperative plasma proteomes of patients with and without POD, (ii) explore key pathophysiological pathways, and (iii) characterise preoperative proteomic profiles to enhance mechanistic understanding of POD.

## Methods

### Study design and population

This investigation is a secondary analysis of the BioCog project (www.biocog.eu)^13^, using an innovative HT technology to assess proteomics^9^. The study included patients aged ≥ 65 years undergoing elective surgery of expected duration of ≥ 60 min with a Mini-Mental State Examination (MMSE) score of ≥ 24 points. Screening included preoperative NCD and frailty^14^. This study was approved by the local Ethics Committee of the Charité Universitätsmedizin Berlin (ref.: EA2/092/14 and 14–469) and was registered at ClinicalTrials.gov (NCT02265263). The study was conducted in accordance with the Declaration of Helsinki as well as local data privacy regulations. Written informed consent was obtained and archived from all patients and/or their legal guardians.

### Postoperative delirium

POD was defined according to the 5th edition of Diagnostic and Statistical Manual of Mental Disorders (DSM-5) criteria^15^. Patients were considered delirious in case of.

- ≥ 2 cumulative points on the Nursing Delirium Screening Scale (Nu-DESC)^16^ and/or a positive Confusion Assessment Method (CAM)^4^ score and/or.
- a positive CAM for the Intensive Care Unit (CAM-ICU)^17^ score and/or.
- patient chart review that showed descriptions of delirium (e.g. confused, agitated, drowsy, disorientated, delirious, antipsychotic therapy).

Delirium screening started in the recovery room and was conducted twice daily (08:00 and 19:00 ± 1 hour) for up to 7 days postoperatively by a research team trained and supervised by psychiatrists and delirium experts, independent of routine hospital procedures.

### Trial design

**Fig. 1:**
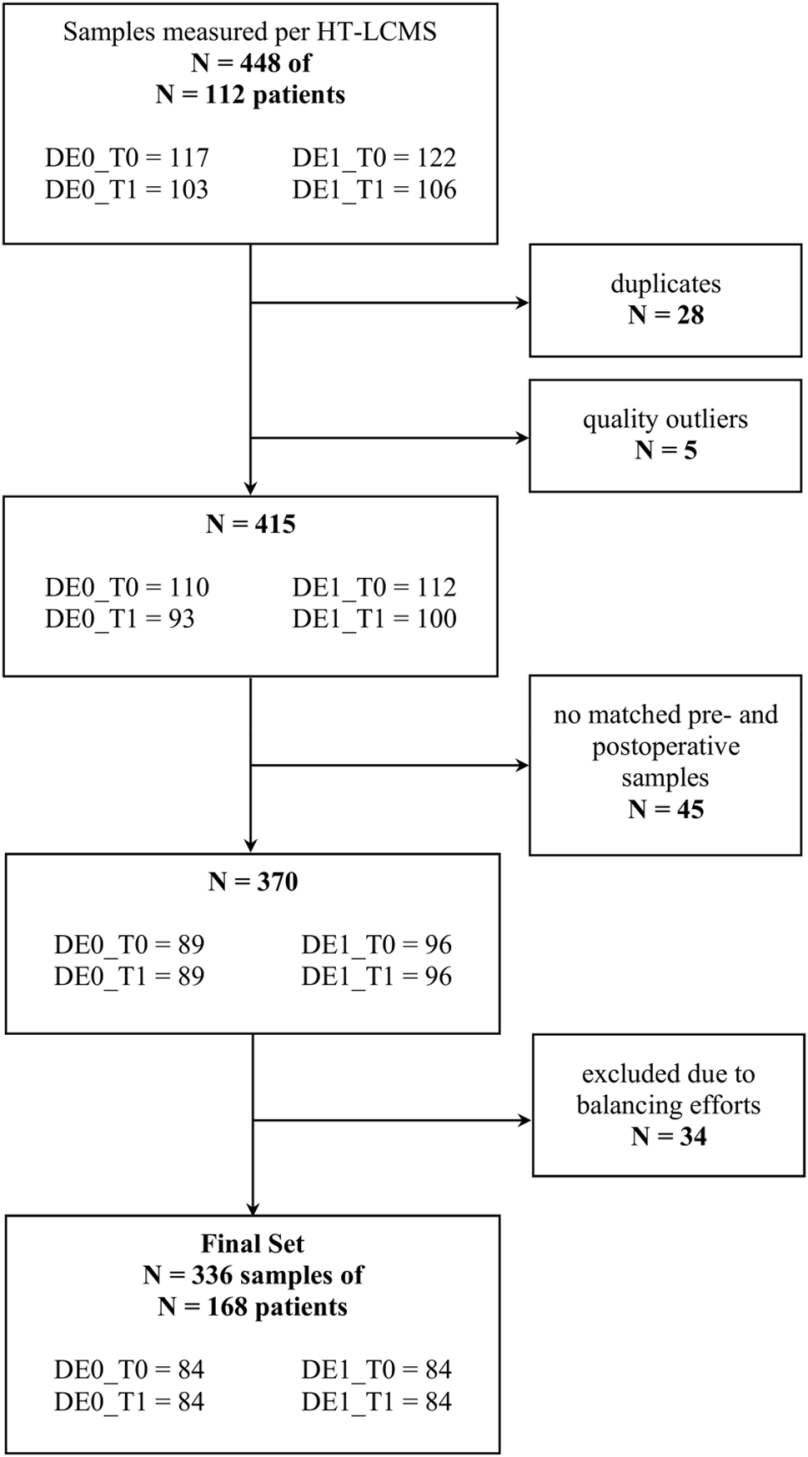
Sample flow chart. The figure shows the selection of samples for analysis. DE0 = non-POD patients, DE1 = POD patients, HT-LCMS = high throughput liquid chromatography mass spectrometry, T0 = preoperative, T1 = postoperative.

At the time of HT proteomic analyses, the BioCog study database was completed and blood samples were stored in the lab facilities of the Charité. We conducted proteomic analyses in a matched case-control design. Given the strong influence of duration of anaesthesia (DoA) on POD^3^, we aimed to match POD cases with a control within a ± 30-minute DoA range or, if impossible, patients were matched iteratively by pairing each case with a control having the closest DoA. The removal of 45 unpaired samples (i.e. no pre- and postoperative sample of the *same* patient) disrupted initial case-control matching. As age and sex can influence proteomic signatures^18^, we rematched samples over sex, age and nutritional status (MNA)^19^, resulting in further removal of 34 samples (17 patients). The final balanced set contained 336 samples (see Fig. 1 and Supplementary Tabs. S1-S6).

### Patient characteristics

The patient cohort was described using physical status according to the American Society of Anaesthesiologists (ASA PS)^20^, Charlson Comorbidity Index (CCI)^21^, grade of NCD and frailty status^14^, impaired activities of daily living according to Barthel (ADL)^22^ and site of surgery.

### Blood sampling

Blood samples were collected preoperatively on the morning of the surgery (T0) and on postoperative day 1 (T1) via venipuncture or arterial cannula. Blood samples were immediately centrifuged, plasma was extracted and frozen in 50 µl aliquots at −80 °C. The samples were thawed for the first time for proteomic analyses, transferred to an Eppendorf twin.tec^®^-PCR plate for proteomic analyses and refrozen at −80 °C.

### Proteomic analysis

To avoid batch effects and confounding of experimental and technical variables, samples were harmonised across plates according to sampling time points. Matched pairs remained together on one plate. Samples were distributed starting with the postoperative samples and then using as many time series as possible. Samples were randomised within plates. Sixteen commercial control samples (12 plasma, 4 serum) were included on each plate to detect sample preparation effects. Semi-automated sample preparation was performed in 96-well format as previously described by Messner et al., using pre-prepared stock solution plates stored at −80 °C^23^. Briefly, 5 µl of thawed plasma samples were transferred to the pre-made denaturation/reduction stock solution plates (50 µl 8 M Urea, 100 mmol ammonium bicarbonate (ABC), 5 µl 5 mmol dithiothreitol) resuspended and incubated at 30 °C for 60 min. 5 µl were then transferred from the iodoacetamide stock solution plate (100 mmol) to the sample plate and incubated in the dark at room temperature for 30 min before dilution with 100 mmol ABC buffer (340 µl). 220 µl of this solution was transferred to the pre-made trypsin stock solution plate (12.5 µl, 0.1 µg/µl) and incubated at 37 °C for 17 h (Benchmark Scientific Incu-Mixer MP4). The digestion was quenched by addition of formic acid (10% v/v, 25 µl) mixture was cleaned using solid phase extraction in C18 96-well plates (BioPureSPE Macro 96-Well, 100 mg PROTO C18, The Nest Group). The eluted samples were vacuum dried and reconstituted in 60 µl 0.1% formic acid with shaking. Insoluble particles were removed by centrifugation and the samples transferred to a new plate. A study pool was generated from all samples. Peptides were resolved on an Agilent 1290 Infinity II (Crick laboratory) in reversed phase mode using a C18 ZORBAX Rapid Resolution High Definition (RRHD) column 2.1 mm x 50 mm, 1.8 μm particles at a column temperature of 30 °C. The eluent was directed to a TripleTOF 6600 mass spectrometer (SCIEX) equipped with IonDrive Turbo V Source (SCIEX) operating in scanning SWATH mode^24^. A linear gradient was applied which ramps from 3% B to 36% B in 5 min (Buffer A: 0.1% FA; Buffer B: ACN/0.1% FA) with a flow rate of 800 µl/minute. For washing the column, the organic solvent was increased to 80% B in 0.5 min and was kept for 0.2 min at this composition before going back to 1% B in 0.3 min. 5 µg of peptide was injected. The DIA/SWATH method consisted of an MS1 scan from m/z 100 to m/z 1500 (20 ms accumulation time) and 25 MS2 scans (25 ms accumulation time) with variable precursor isolation width, covering the mass range from m/z 450 to m/z 850. Ion source gas 1 (nebulizer gas), ion source gas 2 (heater gas) and curtain gas were set to 50, 40 and 25 respectively. The source temperature was set to 450 °C and the ion spray voltage to 5500 V. The study pool was repeatedly injected to monitor LC-MS performance.

### Data preprocessing

The raw proteomics data were processed using DIA-NN 1.8^25^, using standard settings except for MS1 and MS2 resolution which were adjusted to 20 and 12 ppm, respectively, and scan window radius to 6. Peptides were identified with a publicly available plasma library from the DiOGenes study^26^ which was downloaded from the PRIDE repository (PDX013231, accessed on January 19, 2021). Retention times and fragmentation spectra were replaced with in silico generated values by DIA-NN and the peptides were annotated to the Uniprot human canonical proteome with isoform information (3AUP000005640, accessed 20200406) resulting in 2677 protein isoforms, 1530 protein groups and 15 371 precursors. DIA-NN output data matrix of normalised precursor intensities was integrated with metadata. Five samples identified by quality control as outliers were removed, defined as samples having numbers of precursors significantly less than [median − 4.3 x MAD (median absolute deviation)]. Peptides with excessive number of missing values (> 40%) were excluded from our analysis. The missing values of remaining peptides were imputed group-wise (DEk_Tn) using the Principal Component Analysis (PCA) method^27^. After imputation, normalisation of the total dataset was performed using the LIMMA^28^ implementation of cyclic loess method^29^ with option *fast*^30^. It was followed by batch correction using LIMMA^28^. To obtain a quantitative protein data matrix, the log2-intensities of peptides were filtered, only peptides belonging to one protein group were kept, and then summarised into protein log intensity using the “linear models for panel data” method (PLM)^31^ implemented in the preprocessCore R package^32^.

All raw and processed MS data generated in this study have been deposited in the PRIDE repository (ProteomeXchange Consortium) and are publicly available (see section *Data Availability* for details).

### Complementary enzyme activity analysis

In addition to the proteomic approach, plasma butyrylcholinesterase (BCHE) enzyme activity was quantified independently using a point-of-care photometric assay (ChE Check Mobile^®^, Securetec AG, Neubiberg, Germany)^33^ within one hour after sampling.

### Statistical analyses

We took a longitudinal and a cross-sectional approach, both using LIMMA^28^. POD patients are noted as “DE1”, non-POD patients as “DE0”. T0 refers to preoperative, T1 to postoperative day 1. The longitudinal approach used log2 ratios of protein levels at T1 relative to T0 within each group, while the cross-sectional approach analyzed log2 protein expression at individual time points, comparing groups at T0 and T1.

#### Longitudinal approach

Applied model log2[*p*(T1)/*p*(T0)] ~ 0 + Class.

- Contrast 1 – operation effect on non-POD patients – Class = (DE0|T1 – T0).
- Contrast 2 – operation effect on POD patients – Class = (DE1|T1 – T0).
- Contrast 3 – interaction between operation and POD (“pure” POD effect) - (DE1 – DE0|T1 – T0).

In the longitudinal approach we consider log2 ratios to baseline (T0). Therefore, all factors that do not change over time (such as genetic predispositions or consistent baseline protein expression) are canceled out. This provides higher accuracy as compared to the cross-sectional approach.

#### Cross-sectional approach

Applied model log2[*p*] ~ 0 + SubClass.

- Contrast 4 – difference between POD and non-POD patients before operation – (DE1 – DE0|T0).
- Contrast 5 – difference between POD and non-POD patients the day after operation – (DE1 – DE0|T1).
- Contrast 6 – interaction between operation and POD, (“pure” POD effect) – (DE1 – DE0|T1 – T0).

Baseline characteristics are expressed as median (including lower and upper quartile) or as mean (± standard deviation, SD), except for categorical data, which are expressed as frequencies. Differences between groups were tested using Mann-Whitney U test or χ2 test. Regulated proteins were described in fold changes (FC) and considered significant in case of a FC of 1.1 (i.e. log2(1.1) = 0.1375) and significance level of *p* < 0.05 (i.e. -log10(0.05) = 1.30103). FCs are presented in log2(FC), p-values in -log10(p).

As pre- to postoperative BCHE enzyme activity was not normally distributed (Kolmogorov-Smirnov test: *p* = 0.048, Shapiro-Wilk test: *p* < 0.001, Anderson-Darling test: *p* < 0.001), we analysed enzyme activity using the Wilcoxon signed-rank test (two-tailed) with an alpha level of 0.05 (SPSS Statistics, version 30, ©1989, 2024 by SPSS Inc., Chicago, Illinois, USA). A p-value of < 0.05 was considered significant.

### Functional analyses

Gene set enrichment analyses (GSEA) were performed using the clusterProfiler R package^34^ with input derived from statistical contrasts (see above). Results were based on the Reactome Pathway Database (https://reactome.org), applying the Benjamini-Hochberg method for false discovery rate (FDR) correction^35^. To ensure comprehensive coverage, the Gene Ontology Biological Process (GOBP, https://geneontology.org) database was also used, with results included in the Supplementary Material. Pathway enrichment was assessed using the normalised enrichment score (NES).

### Exploratory proteomic classification analysis

Logistic regression (LR) using a generalised linear model from R package caret^36^ was applied to classify patients based on preoperative proteomic profiles. Protein selection was based on LIMMA results for Contrast 4 (α = 0.142 and log2(FC) ≥ 0.1), initially identifying 32 proteins. These candidates were further reduced using recursive feature elimination to maximise receiver operating characteristic (ROC) area under the curve (AUC). The final analysis was based on 8 proteins and employed 5 repeats of 12-fold cross-validation. ROC, AUC, sensitivity, specificity and accuracy were calculated.

## Results

In this matched case-control study we investigated the pre- and postoperative proteomic profile in 168 patients with a 50% POD incidence. Patients were balanced in terms of sex, age, MNA, preexisting NCD and site of surgery. Despite efforts to balance for DoA, there was a significant difference regarding the latter (Table 1 and Supplementary Tabs. S5 and S6). POD patients showed significantly higher ASA PS, CCI scores, impaired daily activities and frailty (Table 1). We identified a total of 226 proteins that were reliably measured in at least 60% of samples in each group. A complete list of all identified proteins, including accession IDs, molecular weights, isoelectric points, numbers of unique peptides/precursors, quantitative statistics and further information is provided as an additional supplementary file (*Supplementary Protein List*).

**Table 1:** Patient characteristics.

Characteristic	POD (DE1) (*n* = 84)	Non-POD (DE0) (*n* = 84)	*p*-value
	*n* = 168		
**Age (years)**	74 [70–76]	73 [69–76]	0.199^a^
**Sex** female	42 (50%)	42 (50%)	1.000
**ASA PS** 1–2 3–4	39 (46.4%) 45 (53.6%)	58 (69.0%) 26 (31.0%)	0.003^b^
**Charlson Comorbidity Index** score (max. 17)	2.10 ± 1.5	1.62 ± 1.7	0.020^a^
**Preexisting NCD** no NCD mild NCD major NCD	49 (66.2%) 16 (21.6%) 9 (12.2%)	50 (70.4%) 12 (16.9%) 9 (12.7%)	0.771^b^
**Frailty Status** robust prefrail frail	27 (37.0%) 24 (32.9%) 22 (30.1%)	38 (63.3%) 13 (21.7%) 9 (15.0%)	0.009^b^
**Impaired ADL**	32 (38.6%)	19 (22.6%)	0.025^b^
**Duration of Anaesthesia (min)**	337 [214; 472]	253 [132; 375]	< 0.001^a^
**Site of Surgery** intracranial intrathoracic/-abdominal/-pelvic peripheral	1 (1.2%) 50 (59.5%) 33 (39.3%)	2 (2.4%) 41 (48.8%) 41 (48.8%)	0.352^b^

Data are expressed as median [25th quartile; 75th quartile], or as mean ± standard deviation except for categorical data, which are expressed as frequencies (i.e. percentages). P-values refer to Mann-Whitney U test (a) or Chi-square test (b) between patients with or without POD. *P* ≤ 0.05 was considered statistically significant. *ADL* activities of daily living, *ASA PS* physical status according to the American Society of Anaesthesiologists, *min* minutes, *NCD* neurocognitive disorder, *SD* standard deviation, *POD* postoperative delirium.

### Statistical analyses

#### Longitudinal approach

**Fig. 2:**
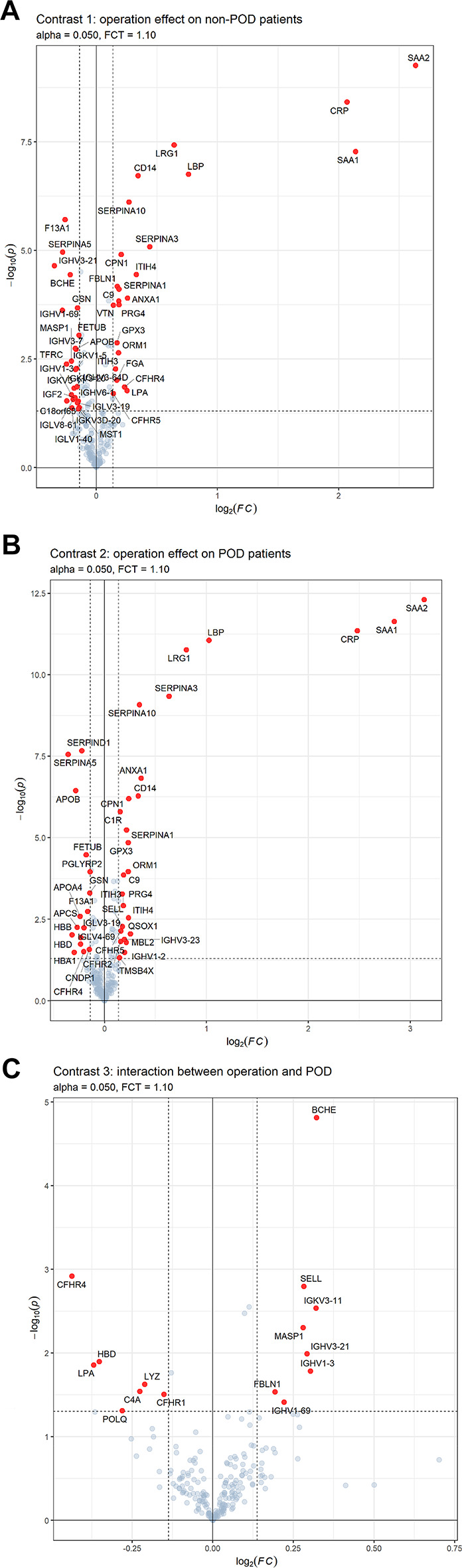
(**a**) Volcano plot for contrasts of protein expression using the longitudinal approach with contrast 1: expression profile regarding operation effect on non-POD patients. Significantly regulated proteins (p < 0.05) are shown in red. Fold change (FC) threshold: 1.1, p-value is displayed in -log10(p), FC in log2(FC). (**b**) Volcano plot for contrasts of protein expression using the longitudinal approach with contrast 2: expression profile regarding operation effect on POD patients. Significantly regulated proteins (p < 0.05) are shown in red. Fold change (FC) threshold: 1.1, p-value is displayed in -log10(p), FC in log2(FC). (**c**) Volcano plot for contrasts of protein expression using the longitudinal approach with contrast 3: expression profile regarding interaction between operation and POD (pure POD effect). Significantly regulated proteins (p < 0.05) are shown in red. Fold change (FC) threshold: 1.1, p-value is displayed in -log10(p), FC in log2(FC).

In the longitudinal approach, we found 45 proteins to be regulated from pre- to postoperative in non-POD patients (Contrast 1) and 42 proteins in POD patients (Contrast 2, Fig. 2a and b). Fifteen proteins are regulated in non-POD patients only (Contrast 1), 8 of which are immunoglobulins (IGs). Fifteen proteins are regulated in POD patients only (Contrast 2). Among them QSOX1, GPX3, MBL2, CFHR2, C1R, TMSB4X are upregulated, and PGLYRP2, CNDP1, SERPIND1, APOA4, APCS, HBA1 and HBB downregulated.

Of special interest are proteins regulated regarding the interaction between operation and POD (Contrast 3), also 15 in total (Fig. 2c). Most of them (except IGHV3-21 and IGHV1-69) have opposite regulation in patients with and without POD (Contrasts 1 and 2), respectively. One protein, CFHR4, is regulated in all three contrasts. Two proteins, SELL, and HBD are significantly regulated only in Contrasts 2 and 3 (SELL is up-regulated in POD patients, while HBD is down-regulated).

A subgroup of interest is regulated in Contrast 1 and 3. While BCHE, MASP1, IGKV3-11, IGHV1-3, IGHV3-21 and IGHV1-69 are downregulated and LPA is upregulated in Contrast 1, they all show opposite regulations in Contrast 3, indicating significance for POD development (see Supplementary Fig. S1 for scatterplot).

#### Cross-sectional approach

**Fig. 3:**
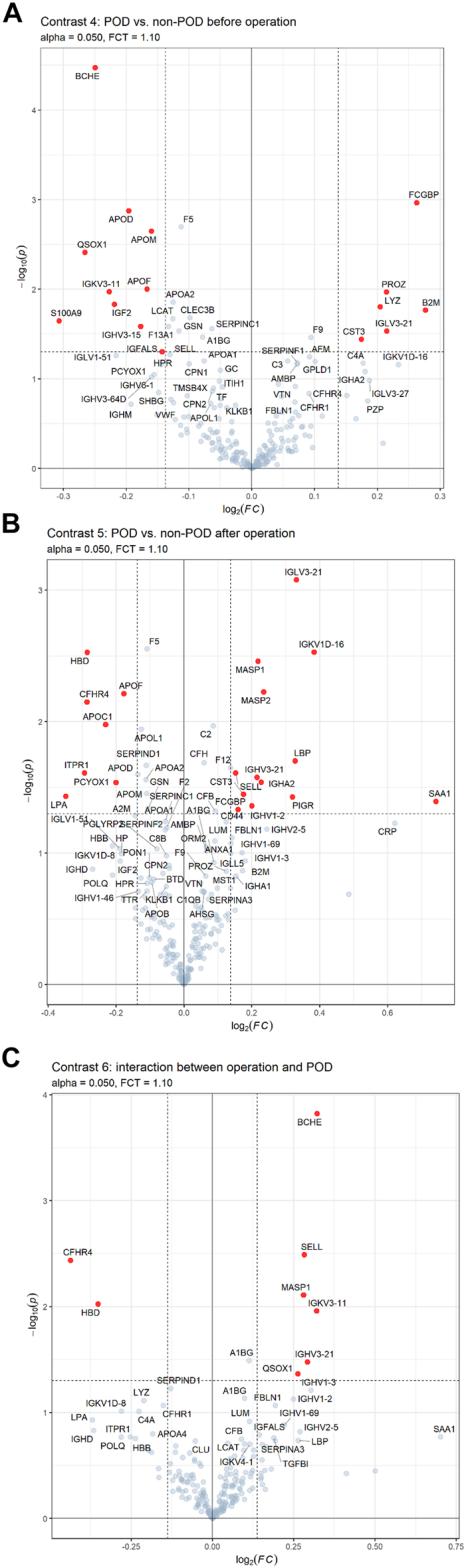
(**a**) Volcano plots for contrasts of protein expression using the cross-sectional approach with contrast 4: expression profile regarding differences between POD and non-POD patients before operation (T0). Significantly regulated proteins (*p* < 0.05) are shown in red. Fold change (FC) threshold: 1.1, p-value is displayed in -log10(p), FC in log2(FC). (**b**) Volcano plots for contrasts of protein expression using the cross-sectional approach with contrast 5: expression profile regarding differences between POD and non-POD patients after operation (T1). Significantly regulated proteins (*p* < 0.05) are shown in red. Fold change (FC) threshold: 1.1, p-value is displayed in -log10(p), FC in log2(FC). (**c**) Volcano plots for contrasts of protein expression using the cross-sectional approach with contrast 6: expression profile regarding interaction between operation and POD (pure POD effect). Significantly regulated proteins (*p* < 0.05) are shown in red. Fold change (FC) threshold: 1.1, p-value is displayed in -log10(p), FC in log2(FC).

The cross-sectional analysis shows a weak POD effect at T0, with Fig. 3a showing only 16 significantly regulated proteins in Contrast 4. The most significant are BCHE (log2(FC) ~ 0.3; FC ~ 1.23) and beta-2-microglobulin (B2M, log2(FC) ~ 0.275; FC ~ 1.21). The effect size at T1 (Contrast 5) is only marginally larger (Fig. 3b). Contrast 6 shows very moderate regulation (Fig. 3c). Scatterplots for Contrasts 4 and 5 are found in Supplementary Fig. S2.

**Fig. 4:**
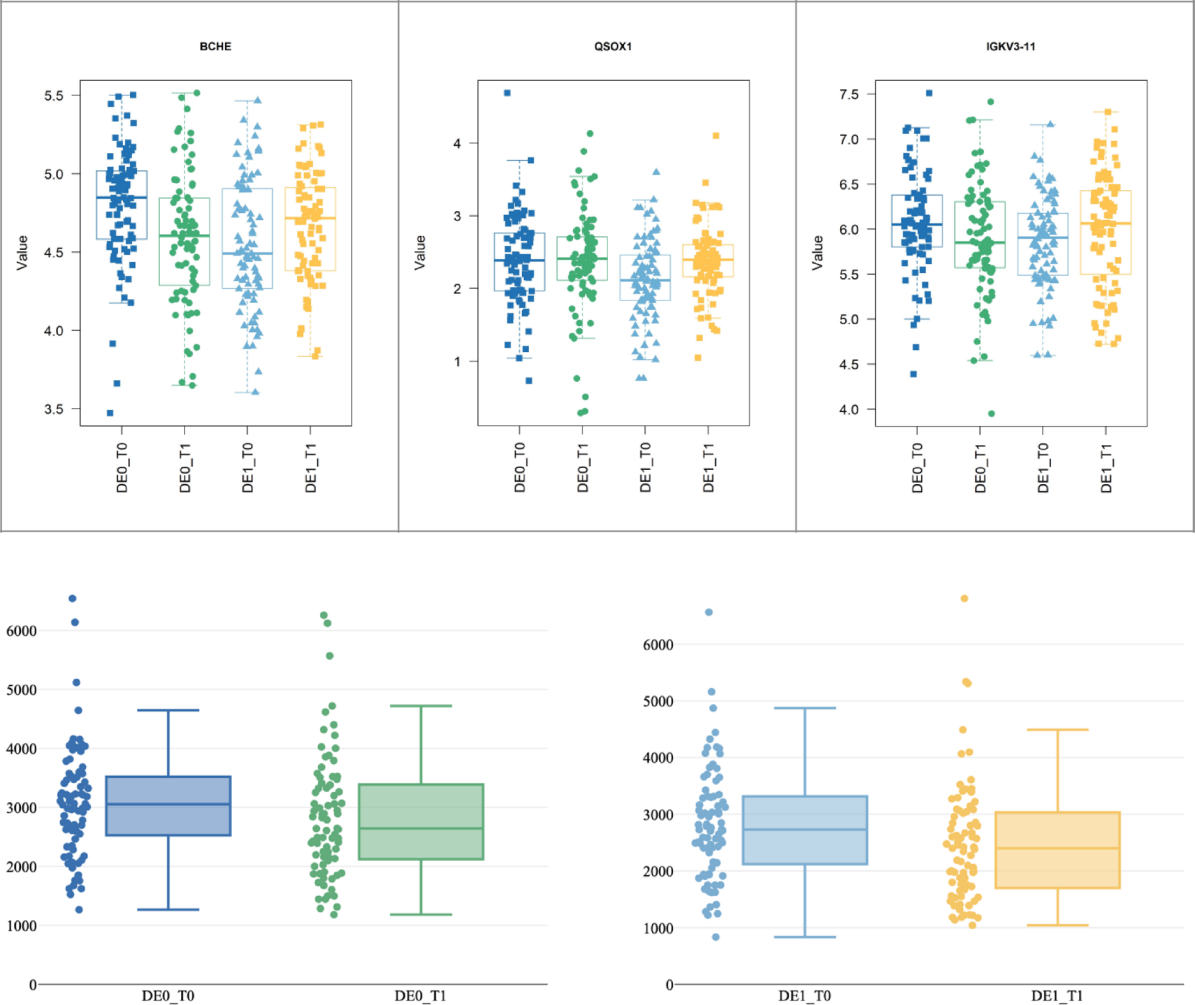
(**a**) Box plots of BCHE, QSOX1 and IGKV3-11 showing preoperative (T0) and postoperative (T1) expression levels (value = log2(Int)) in non-POD (DE0) and POD (DE1) patients. (**b**) Box plots showing butyrylcholinesterase activity (unit: U/l) before (T0) and after (T1) surgery in non-POD (DE0, left panel) and POD patients (DE1, right panel). The median is displayed by the continuous line within the boxes.

BCHE, QSOX1, and IGKV3-11 are present in Contrasts 4 and 6, with lower preoperative expression in POD patients. Postoperatively, their expression increases, reaching levels similar to non-POD patients for QSOX1 and slightly higher for BCHE and IGKV3-11 (see Fig. 4a), explaining their absence in Contrast 5. While BCHE is upregulated in POD patients at T1, analysis of BCHE enzyme activity showed significant postoperative decrease in both groups. In the POD group, 76 of the 84 patients had complete paired data available for analysis. Among these, the median BCHE activity decreased from 2719.85 U/l (IQR: 2119.75–3311.63; 95% CI: 2591.17–3044.91) preoperatively to 2410.46 U/l (IQR: 1737.43–3024.13, 95% CI: 2226.13–2701.90) (Wilcoxon signed-rank test: W = 635.0; *p* < 0.001, *r* = 0.49). In non-POD patients, 78 of 84 patients had complete data. Here, the median BCHE activity significantly decreased from 3052.45 U/l (IQR: 2560.50–3530.13; 95% CI: 2888.73–3216.40) to 2634.40 U/l (IQR: 2102.85–3326.58; 95% CI: 2409.10–2922.20) (W = 784.0, *p* < 0.001, *r* = 0.43). Their respective box plots are shown in Fig. 4b.

Similarly, 9 proteins regulated only in Contrast 4 (e.g. S100A9, IGF2, APOD, LYZ, PROZ, B2M) show a comparable trend without a reversal in regulation. These 12 proteins collectively indicate an interaction with the operation. Five proteins (MASP1, IGHV3-21, SELL, HBD, CFHR4) overlap between Contrasts 5 and 6 (Supplementary Fig. S2), 8 are regulated in Contrast 6.

### Functional analyses

The GSEA results for Reactome are exploratory and complex in nature, here we highlight our key findings.

#### Longitudinal approach

**Fig. 5:**
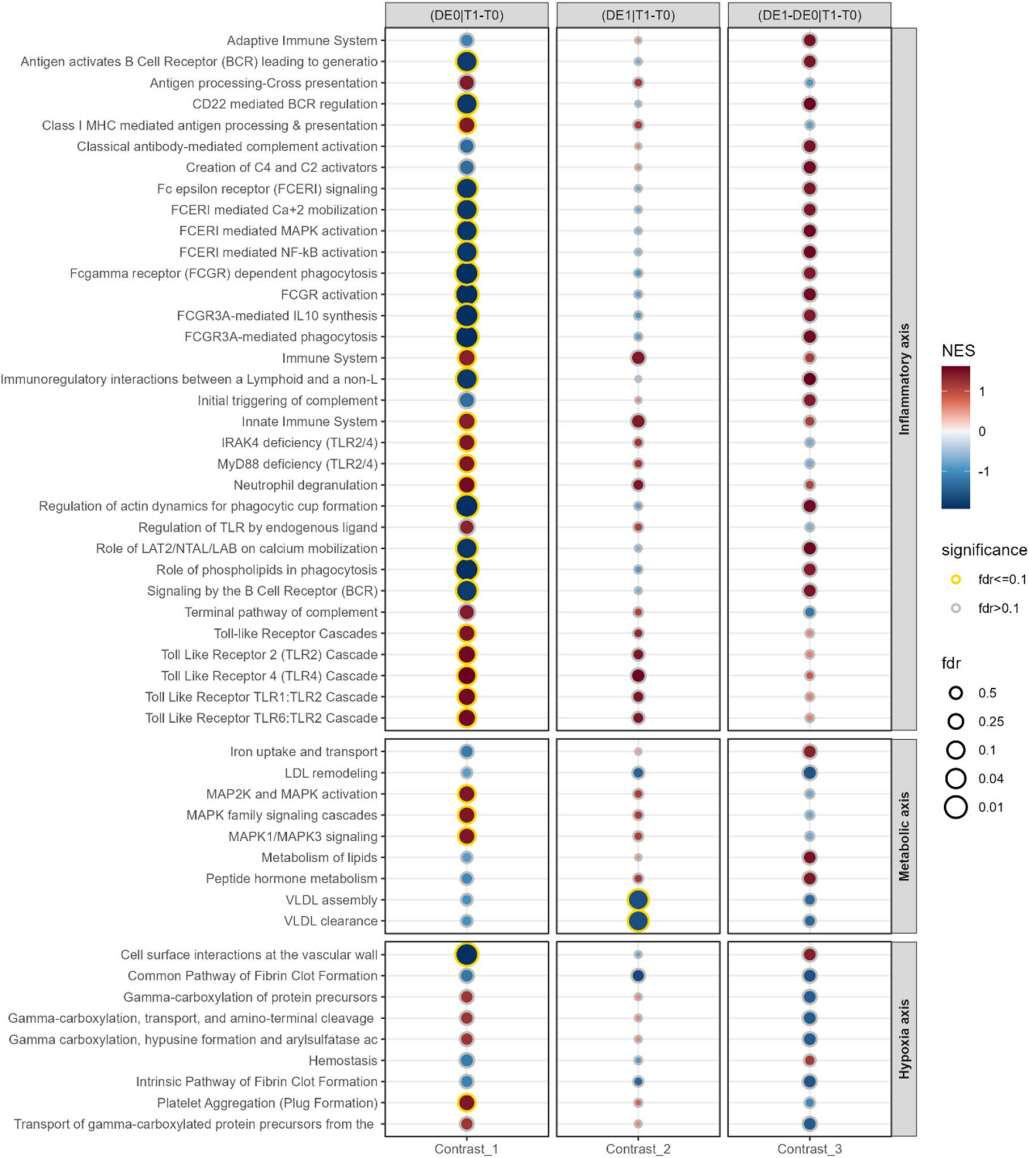
REACTOME pathway enrichment for longitudinal approach (Contrasts 1–3). Pathways with FDR ≤ 0.1 are marked with a golden halo. Pathways are grouped into the pathophysiology domains: Inflammatory axis, hypoxia axis and metabolic axis (see *Introduction*). FDR = false discovery rate, NES = normalised enrichment score. FDR is displayed in -log10(FDR).

Figure 5 shows longitudinal contrasts, highlighting the operation effect. Non-POD patients generally exhibit more significant results by FDR. Among 32 inflammatory/immune-related pathways, we observe complex regulatory patterns. While many pathways, like *Toll-Like Receptor*-associated pathways, show significant upregulation in both cohorts, others are downregulated with a more pronounced effect in non-POD patients (i.e. higher activity in POD patients) with a change of direction in Contrast 3, such as *FCGR/FCERI*-associated pathways. Some display downregulation in healthy patients (C1) and upregulation in both Contrasts 2 and 3, such as *Adaptive Immune System* or 4 *Complement*-related pathways, indicating high activity.

Nine metabolic axis pathways show rather divergent behaviour. In POD patients, *Iron uptake and transport*,* Peptide hormone metabolism* and *Metabolism of lipids* show enrichment in Contrast 3.

Hypoxia/haemostasis axis pathways including 4 *Gamma carboxylation*-related pathways (clotting factor synthesis) and 1 (*Platelet Aggregation*) show strong or weak upregulation in Contrasts 1 and 2, respectively, and a downregulation in Contrast 3. Contrastingly, 2 *fibrin*-pathways exhibit negative enrichment across all contrasts. No toxicity axis-related pathways were observed. For further information, see Supplementary Fig. S3 and Table S7.

#### Cross-sectional approach

**Fig. 6:**
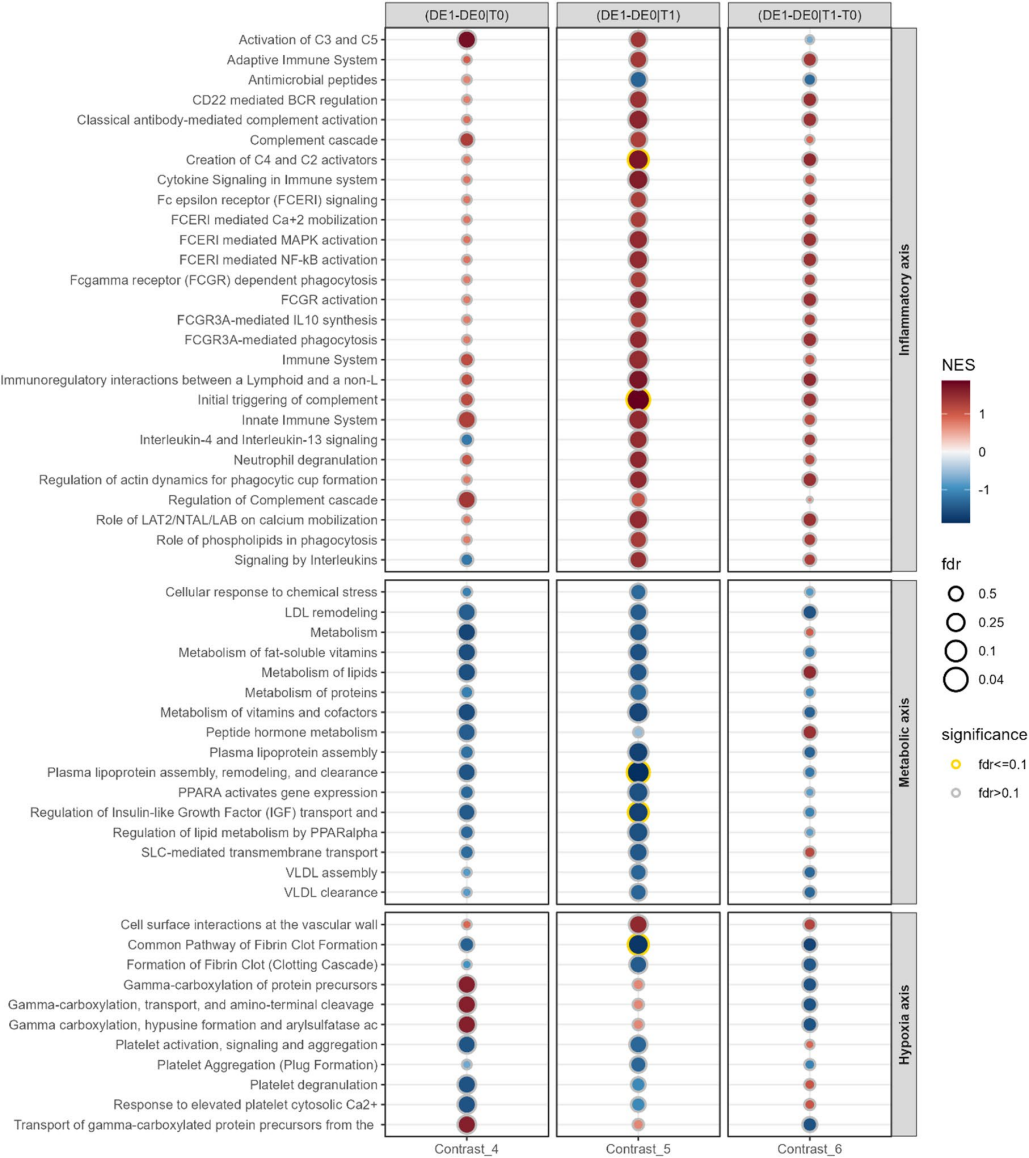
REACTOME pathway enrichment for cross-sectional approach (Contrasts 4–6). Pathways with FDR ≤ 0.1 are marked with a golden halo. Pathways are grouped into the pathophysiology domains: Inflammatory axis, hypoxia axis and metabolic axis (see* Introduction*). FDR = false discovery rate, NES = normalised enrichment score. FDR is displayed in -log10(FDR).

Figure 6 presents cross-sectional contrasts. Significance is generally stronger in Contrast 5. Out of 27 inflammatory axis pathways, 25 demonstrate postoperative enrichment in both groups, with the majority exhibiting greater upregulation in POD patients. Two *Interleukin*-related pathways show postoperative downregulation in healthy patients. Interestingly, 2 complement pathways show stronger enrichment in Contrast 4 (*Activation of C3 and C5*,* Regulation of Complement cascade*).

All 16 metabolic axis pathways show preoperative downregulation in POD patients with only some of them displaying further silencing after surgery. For *Metabolism*, *Metabolism of lipids* and *Peptide hormone metabolism*, we observe downregulation before and after surgery, but upregulation in Contrast 6.

Ten hypoxia axis related pathways show diverse enrichments: whilst *Gamma-carboxylation* pathways are highly enriched before surgery, they are silenced postoperatively. *Platelet*-related pathways show downregulation both pre- and postoperatively, but 3 show activation for pure POD effect. No toxicity axis-related pathways were observed. For further information, see Supplementary Fig. S4 and Table S8.

#### Exploratory proteomic classification analysis

**Fig. 7:**
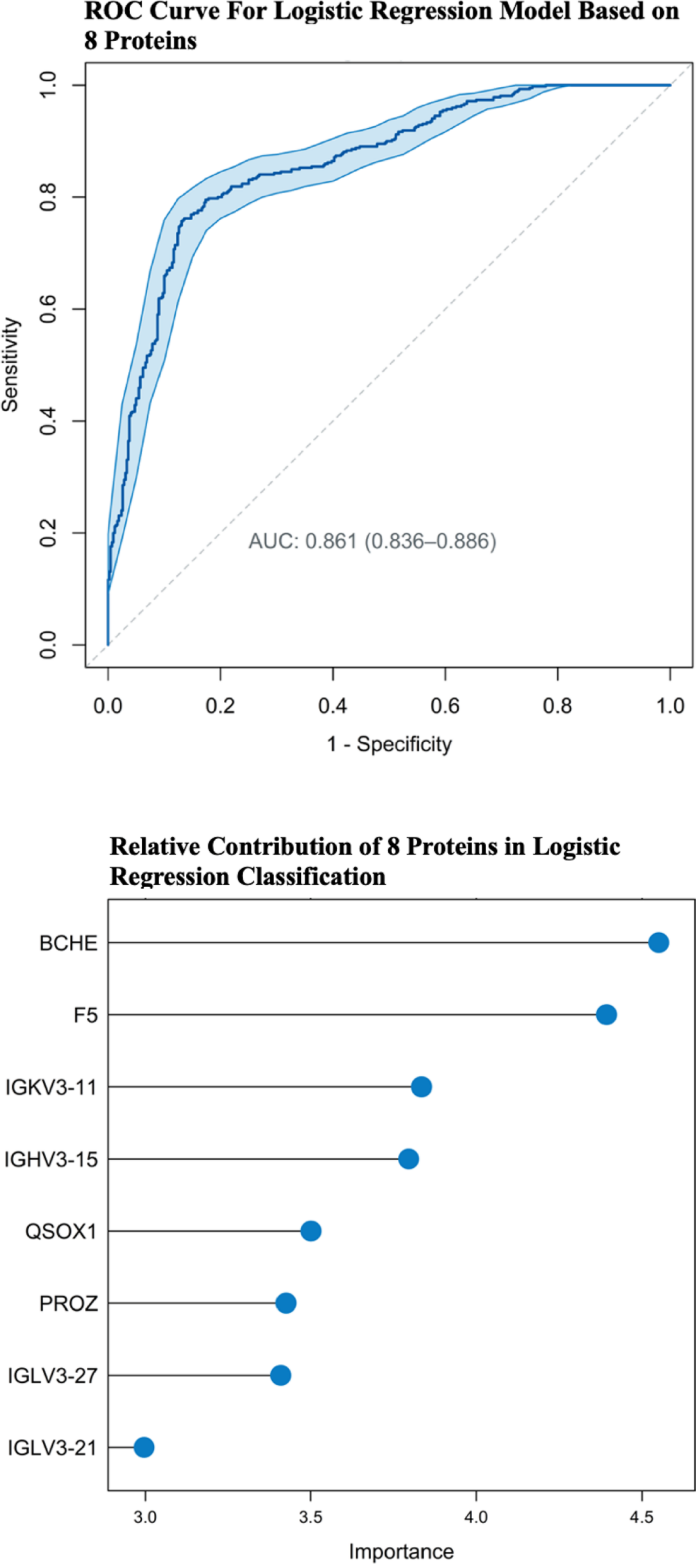
(**a**) Receiver operating characteristics (ROC) of logistic regression (LR) based on preoperative expression profiles (Contrast 4, see *Methods*). (**b**) Feature contribution of the 8 most discriminative proteins identified by logistic regression (LR) based on generalised linear modelling. Importance (x-axis) is displayed based on z-values derived from LR. For further data see also Table S9.

The LR applied to 8 protein expressions showed an AUC of 0.86 with an accuracy of 0.81 (Fig. 7a and b). Sensitivity and specificity were 0.83 and 0.79, respectively. The final list of proteins included BCHE, F5 (factor V), IGKV3-11, IGHV3-15, QSOX1, PROZ, IGLV3-27 and IGLV3-21. For a summary of their respective importance see Supplementary Table S9.

## Discussion

We analysed perioperative proteomics in 168 elderly patients using HT-LCMS, identifying 226 abundant plasma proteins. POD patients had higher (pre-)frailty scores, ASA PS, and CCI classifications, underwent more intracavitary surgeries and experienced prolonged DoA.

This is the first study to apply untargeted proteomics using both longitudinal and cross-sectional designs, integrated with GSEA and preoperative proteomic profiling related to POD, providing mechanistic insights into perioperative vulnerability and identifying pathophysiologic domains and perioperative alterations. This addresses key limitations of prior OMICS studies that have primarily focused on postoperative (i.e. diagnostic) features^12^. While CSF studies may better reflect central nervous system changes due to CSF’s proximity to the brain, plasma sampling is less invasive and may capture alterations secondary to increased BBB permeability in the elderly^12^.

### Longitudinal and cross-sectional approach

We observed an expected increase in acute-phase proteins, including CRP, SAA1/2, and LBP - all of which showed higher expression in POD patients. LBP has been linked to post-stroke delirium^37^, while SAA1/2 can migrate to the brain, enhance neuroinflammation and memory decline and could serve as severity markers for traumatic brain injury^38^. IL-6 is among the most frequently reported proteins in previous studies^12^, however, we did not detect any regulation, possibly due to the absence of pre-analytical immunodepletion. Some authors recommend this step to remove high-abundant proteins (e.g., albumin, IGs), as they can hinder the detection of low-abundant proteins in MS, which includes many POD alterations such as cytokines^12^. MASP1 (mannose-associated serine protease 1) is significantly increased in POD and downregulated in non-POD patients. It activates endothelial cells and initiates the lectin pathway of the complement system by cleaving complement C4 and C2 - a finding confirmed in the GSEA (pathway *Creation of C4 and C2 activators*, Figs. 5 and 6)^39^.

CFHR4 is downregulated in our POD cohort. Functional data is conflicting: mainly its inhibitory effects on the complement system were investigated, whereas new data also indicate activating functions via complex interactions^40^. Another key protein seems to be QSOX1, especially in relation to autophagy, as this process is considered in neuroinflammation^41^. It is downregulated preoperatively, showing enrichment after surgery, but only to the level of non-POD patients, who do not exhibit a significant FC (Fig. 4a). QSOX1 has unclear implications. Some authors find antiapoptotic and protective effects QSOX1-expressing cells as a reaction to oxidative treatment as well as inhibition of autophagy^42^, others demonstrate autophagy stimulation via mitochondrial apoptosis^43^. However, upregulation of QSOX1 seems to be a reaction to oxidative stress rather than a cause^42^.

The most outstanding protein in our analysis is BCHE: it is ubiquitously found, particularly in the liver, blood, pancreas and central nervous system^44^. It serves as the primary acetylcholine (ACH)-hydrolyzing enzyme in human blood and contributes to inflammation and cognitive decline by inhibiting the cholinergic anti-inflammatory pathway^45^. The association between preoperative ChE activity deficits and POD has been documented in several previous studies, such as Bosancic et al.^46^.

We observed a preoperative deficit and postsurgical upregulation in POD patients, while non-POD patients show a postsurgical downregulation. However, this refers to FCs (i.e. relative concentration changes). In contrast, enzymatic activity was significantly reduced in both groups post-surgery. While specific data on this phenomenon are lacking, several mechanisms could be assumed, particularly inflammation and oxidative stress.

BCHE’s apparent divergence between protein abundance and enzymatic activity likely reflects qualitative rather than quantitative alterations, most plausibly arising from post-translational or structural modifications. BCHE is a glycosylated tetramer, and N-glycosylation appears to be its predominant—possibly exclusive—post-translational modification^47^. Oxidative and inflammatory stress can perturb the glycan composition and/or tertiary structure, transiently impairing catalytic efficiency despite unchanged or increased protein abundance. Consistent with this interpretation, BCHE activity inversely correlates with inflammatory markers such as CRP and IL-6^48^. Notably, the known BCHE isoenzymes do not result from alternative mRNA splicing but from genetic polymorphisms, whose distribution remains constant over time within a given cohort^49^. Moreover, the oligomerisation state of BCHE (monomer, dimer or tetramer) does not exert cooperative or activity modulation effects^49^. Hence, neither genetic polymorphisms nor oligomerisation effects seem to account for the observed discrepancy.

Oxidation and nitrosation of cysteine residues increase disulfide bonds and formation of cystine, therefore altering protein stability and function in a powerful manner and nitrosation is caused by the inducible nitric oxide synthase (iNOS), which is induced under inflammatory conditions^50,51^. The correlation between (oxidative) stress and reduced BCHE has been demonstrated in patients with obstructive lung disease^52^.

Other mechanisms of reduced activity may include protein aggregation (but not oligomerisation) and substrate inhibition. The POD-associated FC increase may represent a compensatory mechanism aimed at mitigating preexisting ACh deficits by supplying more substrate as well as compensating for the BCHE activity decline. Of note, astrocytes upregulate BCHE expression under inflammatory conditions mediated by iron dysregulation, resulting in cognitive decline^53^.

In an subanalysis of a different BioCog subcohort (*n* = 127 patients with abdominal surgery, 41% POD incidence), Bosancic et al. observed similar activity (i.e., U/l) declines, although statistically not significant^46^. In the CESARO study, results were comparable to that, with a more pronounced decline in patients > 70 years of age^54^. Postoperative enzyme activity declines in POD patients were also shown in a subanalysis of our DEXDOR trial and further 3 publications in CSF and plasma samples^44,55–57^. The cholinergic system (synthesis, esterase activity, and receptor density) may be influenced by circadian oscillations^58^, but time-of-day effects on BCHE were not systematically examined in this study (see section *Limitations*).

BCHE’s role goes beyond degrading esters. Elevated activity is linked to obesity, metabolic syndrome, chronic kidney disease and diabetes - all of which are linked to chronic inflammation and oxidative stress^58^. Elevated oxidative stress markers, like malondialdehyde or superoxide dismutase, are observed alongside heightened BCHE activity^58^. It plays complex roles in lipid metabolism by production of precursors of LDL and VLDL, resulting in lipid storage and therefore mitochondrial overload and dysfunction^58^.

BCHE regulates energy homeostasis by modulating insulin and glucose levels and by hydrolyzing ghrelin, a multifunctional hormone involved in immune modulation, inflammation, cognitive function and memory^59^. Therefore, a postoperative decrease of ghrelin may alter cognition. However, BCHE’s metabolic implications should be explored by incorporating metabolomics. BBB disruption, influenced by age, oxidative stress and mitochondrial dysfunction, permits complement and immune cell influx, but remains challenging to measure^8,60^ - no such marker was identified in our cohort.

### Pathway enrichment analysis

Our GSEA unravelled complex regulations, most commonly in inflammatory/immune-related pathways, followed by metabolic and hypoxia-related pathways.

#### Inflammatory axis

Both cohorts show postoperative upregulation of numerous pathways within this domain (Contrasts 1–3). However, pathways related to *complement* (2 of 3), *FCERI/FCGR* (8 of 8), and *B cells* (3 of 3) were specifically enriched in POD patients only.

*Complement*-related pathways, including C2, C3, C5 and complement activators, were already slightly elevated preoperatively in patients who later developed POD (Fig. 6, Contrast 4), strongly suggesting a state of low-grade systemic inflammation or immune priming. This particularly applies to the *Activation of C3 and C5* and to the *Regulation of the complement cascade*. Following operation, all but one inflammation pathway showed strong upregulation in POD compared to non-POD patients (Fig. 6, Contrast 5), suggesting that surgical trauma and the subsequent inflammatory response further intensify a preexisting inflammatory susceptibility. We therefore propose an acute-on-chronic disease model. As nearly all inflammatory-axis pathways increased after surgery, however, it remains challenging to pinpoint immune mechanisms specific to POD. Nevertheless, overactivation of complement—particularly C3 and C5—may represent a key driver of pathogenesis.

In contrast, in the longitudinal approach, several *inflammatory axis* pathways showed postoperative downregulation in non-POD patients (Fig. 5, Contrast 1). POD patients, conversely, show the same direction of regulation in 32 of 33 inflammatory pathways (Fig. 5, Contrast 2) but clearly less pronounced than non-POD patients. This indicates that immune activation occurs in both groups, whereas non-POD patients seem to attenuate these physiological responses more effectively, possibly due to lower baseline inflammation.

A small MS–based study in pre- and postoperative immunodepleted CSF revealed pathway enrichment of complement (notably C5) and coagulation cascades^62^. The complement system is known to play an essential role in neurogenesis and synaptic plasticity and to modulate neuroinflammatory states^63^. Our interpretation is further supported by Graves et al.^61^, who demonstrated reduced baseline levels of complement inhibitors C4BPA and CD55 in POD patients by whole-blood RNA transcriptomics. Similarly, Westhoff et al.^62^ proposed that POD may result from a dysfunctional neuroinflammatory response, partly due to reduced concentrations of anti-inflammatory mediators in CSF such as IL-1RA, an observation corroborated by Adamis et al. in plasma^63^.

Taken together, these findings suggest that complement pathways are already primed preoperatively and become further amplified following surgical trauma. As of now, studies of complement inhibition in POD patients are lacking. However, preclinical data in mice examining the overstimulation of complement by administration of exogenous C3a show aggravation of cognitive decline, whereas C3a blockade improved memory performance^64^.

#### Metabolic axis

The longitudinal approach results are heterogeneous. They show strong upregulation of *iron uptake and transport* in POD patients and downregulation in non-PODs. Intracellular iron homeostasis is key for cellular survival - intracellular accumulation leads to ferroptosis, i.e. iron-induced apoptosis via induction of ROS, causing inflammatory cascades and mitochondrial dysfunction^65,66^. It has long been implicated in neuroinflammation and neurodegenerative diseases^65^. Iron overload impairs cellular energy metabolism by hampering the citric acid cycle, increasing anaerobic glycolysis and acidosis^65–67^. The effect of anaesthetics such as sevoflurane, isoflurane and nitrous oxide are complex, numerous and have been described previously: they not only induce ferroptosis but also impede electron transport chain, lead to mitochondrial calcium overload, iron influx imbalance and hamper mitophagia, a process known to degrade dysfunctional mitochondria^68^.

After surgery, POD patients show significantly higher lipid and peptide hormone metabolism than non-POD patients, though only in Contrast 3. Notably, these 2 pathways are among only 4 in the cross-sectional approach that are significantly upregulated in Contrast 6, highlighting their importance. Enhanced lipid and protein metabolism could indicate a compensatory mechanism due to impaired glucose utilisation. All other pathways in the cross-sectional approach show downregulation before and after surgery, implying complex metabolic shutdowns prior to surgical trauma. The pathway *Regulation of IGF transport and uptake by IGFBPs* is of special interest as it is downregulated in POD patients (Contrasts 4–6). Insulin-like Growth Factor 1 (IGF-1) plays a crucial role in the pathophysiology of POD and low levels have been consistently associated with POD^8,11,69^. IGF-1 supports neuronal regeneration, plasticity, synaptogenesis, and BBB integrity, thus mitigating neuroinflammation and autophagy - all factors relevant to POD^70^. Nevertheless, we did not detect specific downregulation of IGF-1 but IGF-2 (significant in Contrast 1 and 4, insignificant in Contrast 5).

#### Hypoxia axis

GSEA results in the coagulation system, being the only pathways identified as enriched, should be interpreted with caution due to divergence. Fibrin clot formation is reduced in all Contrasts. Non-POD patients show stronger enrichment in *Gamma-carboxylation* pathways after surgery, whereas POD patients display a strong enrichment before surgery compared to non-delirious patients, followed by downregulation - a finding of unknown relevance. There is evidence that POD haemostatic dysregulation is more affecting the thrombocytic axis, as 3 of 4 related pathways show enrichment in Contrast 6. Also, there is significant upregulation of *Cell surface interactions at the vascular wall*, supporting thrombocytic interaction with endothelium that might result in microthrombosis as well as proinflammatory signalling^71^. Moreover, thrombocyte activation is a frequent event in sepsis and may play a role in the increased incidence of delirium observed in septic patients^72^. In the review by Wiredu et al., 3 out of the 10 top GO terms from 370 suspected POD markers were *platelet*-associated, all of which show activation in our study. This supports our data quality but also underlines thrombocytes’ involvement in POD^12^. The review covers 8 studies investigating POD-associated proteins in 484 patients and functional analysis of 370 proteins revealed the top 10 GO terms: 3 linked to the immune system, 5 to haemostasis/platelet function and 2 to metabolic pathways^12^. GO terms of the review in relation to our GSEA can be found in Supplementary Table S10. In 8 of the 10 terms we found enrichment in Contrasts 4–6, in one term we found enrichment in Contrasts 1–3 and in one we found upregulation in all Contrasts (*Immune System*). This highlights the concordance of our profiling with previous works and supports current hypotheses that most alterations likely will be found in the domains of *inflammation* and *haemostasis*. Han et al. performed Reactome enrichment of 15 preoperative CSF samples - our analysis is enriched in 7 of their 15 top terms^73^. This suggests certain overlaps between CSF and plasma proteomic signatures, giving reason for parallel CSF and plasma investigation.

### Exploratory proteomic classification analysis

To our knowledge, no proteomic classification approach investigating POD-related profiles has been reported to date^8,12^. However, Tripp et al. described an 11-metabolite signature with an AUC of 0.838, using targeted MS in plasma samples from 52 POD patients in a matched case-control design^74^. Integration of metabolomic and proteomic MS efforts may enhance future biological characterisation of POD-associated patterns. Notably, BCHE levels emerged as a key feature, highlighting the complex involvement of cholinergic pathways.

Remarkably, 4 of the 8 proteins constituting the classifier were variable light or heavy chains of immunoglobulins (IGs). Their levels are highly variable and influenced by demographic and lifestyle factors (e.g., age, sex, BMI, activity^75^. Nevertheless, POD develops in a milieu of systemic inflammation, BBB disruption, and immune/complement activation, where humoral responses are expected to fluctuate and may serve as peripheral proxies of neuroinflammatory risk^76^. FLCs can induce inflammation and may act as immune dysregulation markers, as reported in asthma patients^77^. The enrichment of B-cell-related pathways in POD patients before and after surgery compared to controls (Fig. 6, Contrasts 4–6) supports this interpretation, suggesting that the observed IG signatures could reflect an activated humoral immune response rather than a direct causal mechanism. Contrast 1 shows that non-POD patients downregulate the mentioned pathways, indicating a key role of mitigation of humoral immune response. Similar associations of peripheral B-cell activity with delirium and postoperative neurocognitive changes have recently been reported^78^. Future studies should include targeted IG assays in plasma/CSF and adjust for overall IG or light/heavy-chain levels to control for nonspecific variance.

Factor V (F5) was the second most differentiating protein, with mild but significant downregulation in POD patients. Similarly, Han et al. have shown low F5 levels in preoperative CSF to correlate with POD emergence, suggesting a role in amyloid β destabilisation^73^.

Further classification analyses using linear discriminant analysis, support vector machines, glmnet approaches and neural network modeling did not yield superior discriminative performance compared to LR. The presented classification, derived from a clinically diverse cohort within a matched case-control design, limits extrapolation to general population-based settings. Replication in larger sample batches is required to validate observed mechanistic patterns.

### Strengths and limitations

Our study provides insight into several underlying mechanisms by employing two complementary approaches and GSEA in a contrasting design. It also represents one of the largest case-control cohorts for MS-based POD proteomics to date. However, we acknowledge limitations to our study that preclude broad generalisability across larger populations, including the single-center and retrospective design as well as the matched case–control setup. Despite balancing for major confounders, considerable variability in surgical procedure and exposure to anaesthesia remains, which limits the model’s predictive generalisability. We deliberately avoided restricting the study pipeline to highly specific patient subgroups in order to capture generalised perioperative changes across a broader patient population. Accordingly, the presented classifier should be regarded as hypothesis-generating.

In line with current recommendations for biomarker verification^79^, external validation is underway in an independent, more homogeneous cohort of adult patients (> 18 years) undergoing liver surgery to test the reproducibility of key proteomic signatures and the classifier’s diagnostic accuracy for POD. Preliminary data indicate pronounced age-related differences in proteomic profiles, suggesting that clinical constellations—defined by age, sex, or pre-existing deficits—likely require adapted diagnostic approaches. A subsequent multicenter validation in elderly patients is planned to assess the robustness of the identified signatures across diverse surgical and clinical settings.

We acknowledge that non-standardised sampling time—particularly preoperatively—may have introduced diurnal variability. Baseline samples were collected at varying times before anaesthesia induction, with incomplete timestamp documentation, precluding sensitivity analysis. Postoperative samples were uniformly drawn between 7:00 and 9:00 a.m., minimising circadian effects. The cholinergic system is under circadian control, with ACH release and acetylcholinesterase (ACHE) activity peaking during the active phase and declining during rest^80^; BCHE, however, was not specifically addressed. A recent ICU study reported only mild 24 h fluctuations in serum BCHE, but a light-induced shift^81^. Around 25–30% of the plasma proteome—including inflammatory and complement proteins—show diurnal variation^82^, with C3 and C4 reduced at night^83^. Future analyses will require standardised sampling time.

Finally, no orthogonal, independent affinity-based validation (e.g., ELISA) or absolute quantification of proteins was performed, as antibody availability for most candidates is very limited. Previous proteomic publications working both with CSF and plasma have only partially addressed validation, which was typically limited to a few selected targets such as CRP, AZGP1, SERPINA3, C3 or YKL-40 (CHI3L1)^12,73,84–86^.

## Conclusions

HT-LCMS and functional proteomics are valuable tools for investigating pathophysiological mechanisms underlying POD, with the potential to (i) enhance our comprehension of its complex pathogenesis, (ii) generate novel mechanistic hypotheses and (iii) identify proteomic patterns associated with perioperative vulnerability. Incorporating additional OMICS approaches, particularly metabolomics, may enrich mechanistic insights. Our findings require reproduction in larger, independent cohorts and secondary validation by orthogonal technologies to strengthen the biological relevance of the observed patterns.

## Supplementary Information

Below is the link to the electronic supplementary material.

Supplementary Material 1

Supplementary Material 2

## Data Availability

The complete set of mass spectrometry proteomics data generated in this study has been deposited to the ProteomeXchange Consortium via the PRIDE partner repository. The dataset was uploaded as partial submission containing all files to reproduce the analysis: raw data files, spectral library, fasta file, the results/quantitative output tables (DIA-NN), and the accompanying metadata and README files describing the sample-to-file mapping, acquisition parameters, and data processing workflow. The dataset has been assigned the identifier PDX071265. Further clinical data supporting the findings of this study are available from the corresponding author upon reasonable request.During the peer-review process, the dataset can be accessed by logging in to the PRIDE website using the following details: Project accession: PXD071265; Token: yN0dMZHOJknWAlternatively, reviewers can access the dataset by logging in to the PRIDE website using the following account credentials: Username: reviewer_pxd071265@ebi.ac.uk; Password: oWzv0lKeRtuc.

## Abbreviations

ABC: Ammonium bicarbonate
ACH: Acetylcholine
ACHE: Acetylcholine esterase
ADL: Activities of daily living
APCS: Serum amyloid P component
APOA4: Apolipoprotein A-IV
APOF: Apolipoprotein F
ASA PS: American Society of Anaesthesiologists physical status
AUC: Area under the curve
AZGP1: Alpha-2-glycoprotein 1, zinc-binding
B2M: Beta-2-microglobulin
BBB: Blood-brain barrier
BCHE: Butyrylcholinesterase
BMI: Body mass index
C18orf63: Chromosome 18 open reading frame 63
C1R: Complement component 1, R subcomponent
C2: Complement component 2
C3(a): Complement component 3 (activated)
C4: Complement component 4
C4BPA: Complement component 4 binding protein alpha
C5: Complement component 5
CAM-ICU: Confusion assessment method for the intensive care unit
CCI: Charlson comorbidity index
CD55: Complement decay-accelerating factor (DAF)
CFHR2: Complement factor H-related protein 2
CFHR4: Complement factor H-related protein 4
ChE: Cholinesterase
CHI3L1: Chitinase-3-like protein 1 (also known as YKL-40)
CNDP1: Carnosine dipeptidase 1
CRP: C-reactive protein
CSF: Cerebrospinal fluid
CST3: Cystatin C
DE0: Non-POD patients
DE1: POD patients
DIA-NN: Data-independent acquisition neural networks
SWATH: Sequential window acquisition of all theoretical mass spectra
DoA: Duration of anaesthesia
DSM-5: Diagnostic and Statistical Manual of Mental Disorders, 5th edition
EEG: Electroencephalogram
ELISA: Enzyme-linked immunosorbent assay
F5: Factor V
FBLN1: Fibulin 1
FC: Fold change
FCGBP: Fc fragment of IgG binding protein
FCGR: Fc gamma receptor
FCERI: Fc epsilon receptor I
FDR: False discovery rate
FGA: Fibrinogen alpha chain
GO: Gene Ontology
GOBP: Gene Ontology Biological Process
GPX3: Glutathione peroxidase 3
GSEA: Gene set enrichment analysis
HB: Haemoglobin
HBA1: Haemoglobin subunit alpha 1
HBD: Haemoglobin subunit delta
HT-LCMS: High-throughput liquid chromatography-mass spectrometry
IG: Immunoglobulin
IGF-1: Insulin-like growth factor 1
IGF2: Insulin-like growth factor 2
IGFALS: Insulin-like growth factor binding protein, acid-labile subunit
IGFBPs: Insulin-like growth factor binding proteins
IGHV: Immunoglobulin heavy variable
IGKV: Immunoglobulin kappa variable
IL: Interleukin
IL-1RA: Interleukin-1 receptor antagonist
iNOS: Inducible nitric oxide synthase
LBP: Lipopolysaccharide-binding protein
LPA: Lipoprotein(a)
LR: Logistic regression
LYZ: Lysozyme
MAD: Median absolute deviation
MASP1: Mannose-associated serine protease 1
MBL2: Mannose-binding lectin 2
MMSE: Mini-mental state examination
MNA: Mini nutritional assessment
MST1: Macrophage stimulating 1
NCD: Neurocognitive disorder
NES: Normalised enrichment score
Nu-DESC: Nursing delirium screening scale
PCA: Principal component analysis
PGLYRP2: Peptidoglycan recognition protein 2
PLM: Panel linear model
POCD: Postoperative cognitive dysfunction
POD: Postoperative delirium
ppm: Parts per million
PROZ: Protein Z
QSOX1: Quiescin sulfhydryl oxidase 1
RNA: Ribonucleic acid
ROS: Reactive oxygen species
S100A9: S100 calcium-binding protein A9
S100B: S100 calcium-binding protein B
SAA1: Serum amyloid A1
SD: Standard deviation
SELL: L-selectin
SERPINA3: Serpin family A member 3 (alpha-1-antichymotrypsin)
SERPIND1: Serpin family D member 1
SWATH: Sequential window acquisition of all theoretical mass spectra
T0: Preoperative time point 0 (baseline)
T1: Postoperative time point 1 (day 1)
TFRC: Transferrin receptor
TMSB4X: Thymosin beta-4 X-linked
TNF-ɑ: Tumor necrosis factor-alpha
VTN: Vitronectin
